# Enhancing or Inhibitory Effect of Fruit or Vegetable Bioactive Compound on *Aspergillus niger* and *A. oryzae*

**DOI:** 10.3390/jof8010012

**Published:** 2021-12-24

**Authors:** Gülru Bulkan, Sitaresmi Sitaresmi, Gerarda Tania Yudhanti, Ria Millati, Rachma Wikandari, Mohammad J. Taherzadeh

**Affiliations:** 1 Swedish Centre for Resource Recovery, University of Borås, 50190 Boras, Sweden; Mohammad.Taherzadeh@hb.se; 2 Department of Food and Agricultural Product Technology, Faculty of Agricultural Technology, Universitas Gadjah Mada, Yogyakarta 55281, Indonesia; sitaresmi@mail.ugm.ac.id (S.S.); gerardatania@mail.ugm.ac.id (G.T.Y.); ria_millati@ugm.ac.id (R.M.); rachma_wikandari@mail.ugm.ac.id (R.W.)

**Keywords:** filamentous fungi, fruit processing waste, *Aspergillus*, bioactive, flavor, inhibitor

## Abstract

Fruit and vegetable processing wastes are global challenges but also suitable sources with a variety of nutrients for different fermentative products using bacteria, yeast or fungi. The interaction of microorganisms with bioactive compounds in fruit waste can have inhibitory or enhancing effect on microbial growth. In this study, the antimicrobial effect of 10 bioactive compounds, including octanol, ellagic acid, (−)-epicatechin, quercetin, betanin, ascorbic acid, limonene, hexanal, car-3-ene, and myrcene in the range of 0–240 mg/L on filamentous fungi *Aspergillus* *oryzae* and *Aspergillus niger* were investigated. These fungi were both found to be resistant to all compounds except octanol, which can be used as a natural antifungal agent, specifically against *A. oryzae* and *A. niger* contamination. On the contrary, polyphenols (quercetin and ellagic acid), ascorbic acid, and hexanal enhanced *A. niger* biomass yield 28%, 7.8%, 16%, and 6%, respectively. Furthermore, 240 mg/L car-3-ene was found to increase *A. oryzae* biomass yield 8%, while a 9% decrease was observed at lower concentration, 24 mg/L. Similarly, up to 17% decrease of biomass yield was observed from betanin and myrcene. The resistant nature of the fungi against FPW bioactive compounds shows the potential of these fungi for further application in waste valorization.

## 1. Introduction

The increasing population of the earth and its demands, such as energy and industrialization, result in residual waste material that pollutes environment. The longstanding trend of linear economy can be changed to a circular economy in which the industrial by-products/residuals can be valorized and re-gained into the market instead of being wasted [1]. The fruit-processing industry generates more than 0.5 billion tones waste [2]. Fruits are processed to produce juice, cider, dried products, etc. [1] and result in residuals, such as orange peel, mango peel and seed, pomegranate peel, apple pomace, etc. [1,2,3]. Fruit-processing waste (FPW) is utilized for animal feed, biogas, or biofertilizer production [2,4]; however, in many cases, it ends up in landfills, and disposal can be costly within environment protection regulations [2]. The high moisture content of the material decreases the life span and limits its applications since it can easily degrade [2]. Therefore, there are various studies on the valorization of FPW, e.g., biodegradable packaging material [5], biocomposite [6], bioactive compound extraction [7,8], biogas production, ethanol production, etc. The material can be valorized through microorganisms that have the ability to secrete diverse enzymes, i.e., filamentous fungi.

Filamentous fungi are wide-spread microorganisms in nature and an essential part of ecosystem with their capability of decomposing organic materials. Imitating nature, they can take place in bioconversion of waste streams into value-added products. In comparison to other microorganisms, filamentous fungi can be more beneficial in waste valorization. They can grow on various material, and the biomass can be separated energy-efficiently due to their filamentous growth [9]. *Aspergillus* genus, a member of Ascomycete filamentous fungi [10], are known for their wide variety of enzymes, which allow them to degrade and convert various organic materials into metabolites and biomass [11,12,13]. Their high capability for producing extracellular enzymes (cellulase, pectinase, amylases, xylanases, etc.) makes them interesting for the industry [14,15]. While some *Aspergillus* strains are defined as GRAS (Generally Recognized As Safe) [16], some others are reported to be pathogenic due to the mycotoxins they produce [17,18]. Among *Aspergillus* genus, *Aspergillus oryzae*, which is traditionally used in Southeast Asian cuisine for soy sauce, miso, sake, etc. [19], and *Aspergillus niger*, a pathogenic filamentous fungus, are two widely used fungi in the industry [19,20]. *A. oryzae* is capable of producing organic molecules, such as citric acid [14], glutamic acid [21], ethanol [22], and various enzymes important in food and detergent industry, i.e., glucoamylase, α-amylases, cellulase, and proteases, etc. [21]. *A. niger* is also a significant source of various organic acid and enzymes, such as citric acid, gluconic acid [23], pectinase, protease, amyloglucosidase [23], exoglucanase, and xylanase [14]. Filamentous fungi bioprocesses provide multiple products, such as enzymes, organic acids, biomass, ethanol, etc. These multiple products from a single source provide economic benefits to fungal biorefineries.

FPW contains fruit flavors, which are compounds that create the unique taste of the fruit and vegetables [24,25]. Fruit flavors are a combination of “sugars, salts, acids, bitter compounds such as alkaloids and flavonoids, and aroma volatiles” [26,27]. These chemically varying compounds, such as “alcohol, aldehyde, ketone, lactone, terpenoid, and ester” [25,28] are produced during fruit maturation [25]. In addition to their health-benefitting effects on human, such as anti-oxidative and anti-inflammatory properties [29], they have crucial functions for fruit, such as protection against UV light, climate conditions (frost, drought, etc.), signal molecules, detoxifying agents, and antimicrobial compounds [29]. The compounds exist in trace amounts in various parts of the fruit, including edible parts and those discarded in the industry, such as peels and seeds [2,30], which is also convenient to protecting fruits from microbial decay [25]. Villacís-Chiriboga et al. [30] stated that the residual parts contain more bioactive compound than the edible part.

Valorization of FPW by microbial processes can be challenging due to the inhibitory effect of existing flavor compounds (FC). Microbial culture (bacteria, archaea, yeast, filamentous fungi) can be used to convert the residual materials (carbohydrates, proteins, etc.) into value-added products, such as enzymes, proteins, cell biomass, and chemicals (i.e., biomethane, bioethanol). Wikandari [25] studied the inhibitory effect of various FC on bacteria and biogas production. Among several FC that were investigated, epicatechin, quercetin, car-3-ene, and hexanal were moderate inhibitors on anaerobic digestion, while myrcene was the strongest, followed by octanol. The methane production decreased 50% when myrcene concentration was less than 0.005%. Similarly, Panyadee et al. [31] studied methane production from *Phyllanthus emblica* residues. It was stated that the tannins existing in *Phyllanthus emblica* residues (which mainly degrade into gallic acid, ellagic acid, and glucose) have a negative effect on biogas production, and biogas production was improved in the case of co-digestion with food waste. In other studies, the inhibiting effect of limonene in citrus waste and the need for limonene removal for methane production was stated [32,33]. The obstacle with the inhibitory effect of FC might be overcome by using other microorganisms, such as filamentous fungi. Unlike bacteria, there is a scarcity of information about the inhibitory effect of FC on filamentous fungi.

The aim of this study is to investigate the effect of flavor compounds (FC) on filamentous fungi. Since the most widespread fresh fruit-degrading fungi among *Aspergillus* species is reported to be *A. niger* [18], in this study, *A. niger* was tested against 10 bioactive compounds (octanol, ellagic acid, (−)-epicatechin, quercetin, betanin, ascorbic acid, limonene, hexanal, car-3-ene, and myrcene) available in the 0.006–248 mg/kg concentration range in fruit-processing residuals [34,35,36,37,38,39,40]. As an alternative to *A. niger*, the edible strain *A. oryzae* was also studied in terms of its resistance and growth potential for further valorizations of FPW.

## 2. Materials and Methods

### 2.1. Microorganisms

Two different *Aspergillus* strains, one with edible and one with pathogenic properties, were used. The edible *Aspergillus oryzae* var. *oryzae* CBS 819.72 (Centraalbureau voor Schimmelcultures, Utrecht, the Netherlands) and *Aspergillus niger,* which was isolated as a contaminant from the experimental studies in our research group, were used. All fungi were grown on potato dextrose agar (PDA) agar plate containing 20 g/L glucose, 15 g/L agar, and 4 g/L potato extract [22]. The agar plates were kept in an incubator for 3 days at 30 °C. For preservation, they were kept at 4 °C, and new plates were prepared after 1 month. The spores grown on the agar plate were collected via adding 20 mL sterile distilled water into the agar plate and collecting the spore solution after mixing the water with spores using a L-shaped spreader. 

### 2.2. Cultivation Medium and Chemicals

Shaking flask cultivations were carried out in a 250-mL cotton-plugged Erlenmeyer flask containing 100 mL of synthetic medium. The synthetic medium consisted of 30 g/L glucose, 7.5 g/L (NH_4_)_2_SO_4_, 3.5 g/L KH_2_PO_4_, 2.2 g/L CaCl_2_·2H_2_O, 1 mL/L vitamin solution, and 10 mL/L trace metal solution, according to [41]. The initial pH was adjusted to 5.5, and pH was controlled during the cultivation at 35 °C, with a 125 rpm shaking water bath. The spore solution added to each flask was 1 mL/50 mL medium. The spore concentration was 1.5–2.2 × 10^7^ spores/mL for *Aspergillus oryzae* and 1.6–4.1 × 10^7^ spores/mL for *Aspergillus niger.*

The flavor compounds used in this study were purchased from Sigma Aldrich (St. Louis, MO, USA), except D-limonene (96%), which was purchased from Acros Organic, NJ, USA. The bioactive compounds used in this study were D-limonene, myrcene, car-3-ene, quercetin, epicatechin, ellagic acid, octanol, betanin, hexanal, and L-ascorbic acid. Stock solution of each chemical (0.5% *w*/*v*) was prepared by dissolving the flavor compound in the appropriate solvent. The flavor compounds and the corresponding solvent solutions are shown in Table 1. For limonene, quercetin, and epicatechin, higher concentration solutions (10% limonene (*w*/*v*) in absolute ethanol, 10% (*w*/*v*) quercetin in absolute ethanol, 1% (*w*/*v*) epicatechin in 20% aqueous ethanol) were prepared initially due to their low solubility in miliQ water. Then, these solutions were diluted to the concentration of 0.5% (*w*/*v*) flavor compound. Three different concentrations of each flavor compound were prepared: 0.5% (*w*/*v*), 0.05% (*w*/*v*), and 0.005% (*w*/*v*). The solvent concentration of 0.05% (*w*/*v*) and 0.005% (*w*/*v*) flavor compound solutions was also adjusted to the solvent level of 0.5% (*w*/*v*) solution. From each concentration, 5 mL of solution was added into 100 mL synthetic medium. The range of tested concentrations were adjusted considering the actual concentrations in fruit/fruit-industry residuals. In a control flask, 5 mL of miliQ water was added to complete the volume. For the compounds that were dissolved in a solvent other than water (except ellagic acid, which had only miliQ added into synthetic medium as control), another control was prepared, and the amount of residual solvent in the cultivation medium was added into the control medium in order to observe the sole effect of the flavor compound.

### 2.3. Analytical Methods

Biomass produced during cultivation was separated using a sieve. After washing with distilled water, it was dried at 70 °C oven. Biomass production was reported as g/L, and the biomass yield was reported as g biomass/g initial glucose. The samples taken during 72-h cultivation were analyzed using high-performance liquid chromatography (HPLC). The column used in HPLC was a hydrogen-ion-based ion-exchange column (Aminex HPX-87H, Bio-Rad, Hercules, CA, USA) at 60 °C, and the eluent was 0.6 mL·min^−1^ 5 mM H_2_SO_4_. Glucose, glycerol, ethanol, acetic acid, and lactic acid were analyzed using the HPLC.

### 2.4. Statistical Analysis

All experiments were carried out as duplicate. The standard deviation is shown in graphs with 95% confidence interval. The data were analyzed with one-way ANOVA using MINITAB software (Minitab Ltd., Coventry, UK) (*p* < 0.05).

## 3. Results and Discussion

Fruits get their unique taste and properties from the flavor compounds they have. Apart from imparting the taste of the fruit, some flavor compounds take part in the defense mechanism of the plant against microbial decay [27]. Additionally, some of these bioactive compounds show health-improving effects with their antioxidant properties. The bioactive compounds produced by plants end up in nature in the case of incorrect handling. This situation results in microbial growth creating an environment susceptible to diseases. Moreover, bioactive compounds leakage to environment can cause a negative effect on natural life in case of accumulation. Some of the compounds considered in this study are also considered as emerging organic contaminants, such as car-3-ene and limonene [42,43]. However, some microorganisms, such as filamentous fungi, belong to Ascomycota and Basidiomycota and are capable of converting emerging organic contaminants [44].

**Figure 1:**
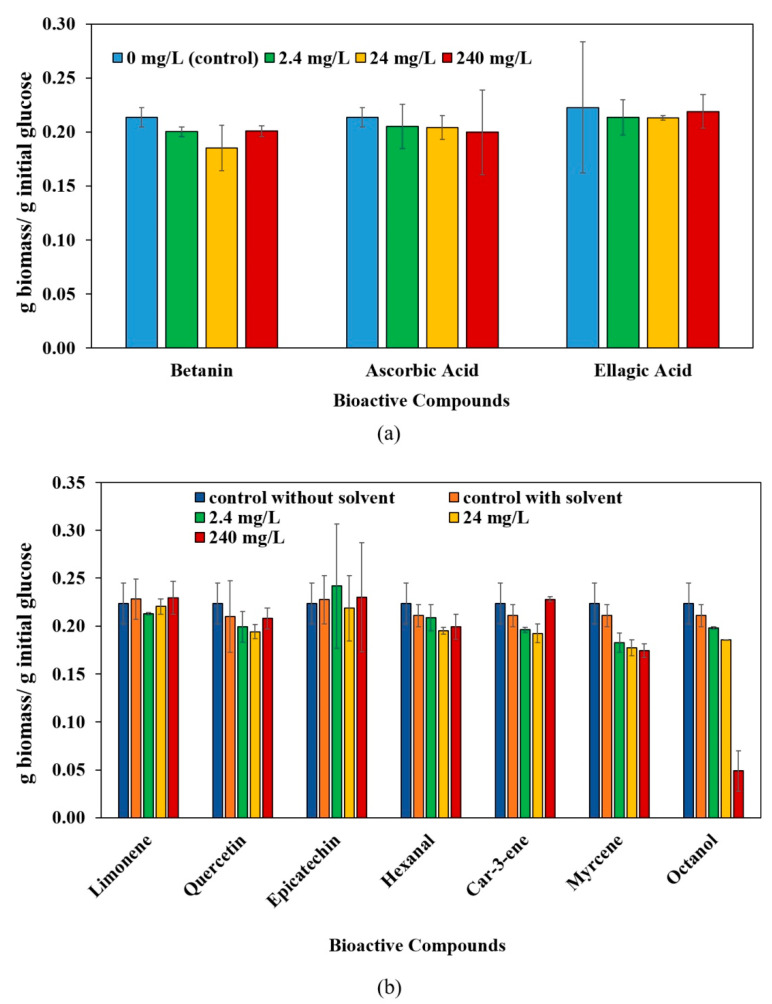
The effect of FPW bioactive compounds on biomass yield of *A. oryzae*. (**a**) The compounds that had water as solvent (ellagic acid had NaOH solution as a solvent; however, since the initial pH of the medium was at the required pH of cultivation, the control condition was the same as betanin and ascorbic acid). (**b**) The compounds that had a solvent other than water had two control (one control without bioactive compound and solvent and another control with solvent without bioactive compounds).

**Figure 2:**
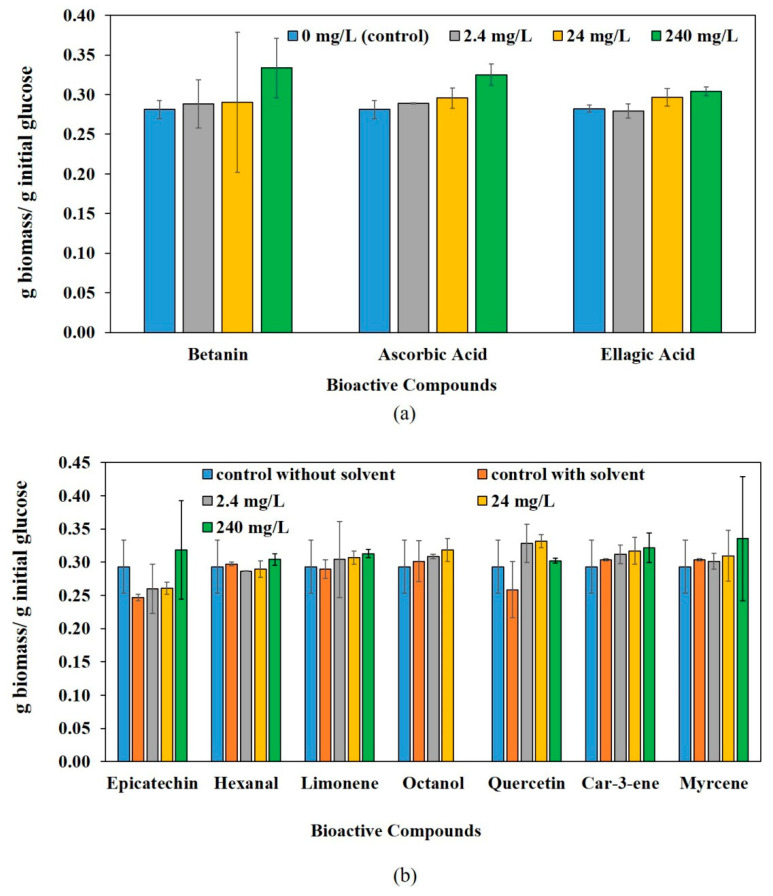
The effect of bioactive compounds on *A. niger* biomass yield. (**a**) The compounds that had control without bioactive compound and solvent (ellagic acid had NaOH solution as a solvent; however, since the initial pH of the medium was at the required pH of cultivation, the control condition was the same as betanin and ascorbic acid). (**b**) The compounds that had a solvent other than water had two control (one control without bioactive compound and solvent and another control with solvent and without bioactive compounds).

In this study, 10 different bioactive compounds were investigated regarding their effect on filamentous fungi, particularly *Aspergillus oryzae* and *Aspergillus niger.* These compounds exist in variety of fruits, such as citrus, mango, cashew apple, peach, pomegranate, apple, plum, redbeet (beetroot), pitahaya, grape, and various types of berries [8,25,45,46,47,48]. The concentration range used in this study was 0–240 mg/L, based on concentration range 0.006–248 mg/kg found in fruit-processing residues in literature. The effect on biomass yields of *A. oryzae* and *A. niger* are shown in Figure 1 and Figure 2, respectively.

For each flavor compound, the effects of different concentrations were compared to each other and their respective control with solvent. Betanin, ascorbic acid, and ellagic acid were compared to control, which had no solvent different than water. The remaining compounds, shown in Figure 1b and Figure 2b, had a different solvent than water in their stock solution (Table 1). Hence, they had a second control (with solvent and without bioactive compound) at the same solvent concentration as the medium containing bioactive compounds. The existence of bioactive compounds was the only difference between the second control medium and the medium containing bioactive compounds. Therefore, the statistical comparison was done by comparing the control (with solvent and without bioactive compound) and different concentrations. The *p*-value results lower than 0.05 are shown in Table 2 and Table 3 for *A. oryzae* and *A. niger*, respectively. 

### 3.1. Ellagic Acid

The growth of *A. oryzae* and *A. niger* used in this study were not negatively affected by existence of ellagic acid in the cultivation medium. For *A. niger*, the highest concentration of ellagic acid showed slightly enhancing effect on biomass yield, 0.30 ± 0.01 g biomass/g initial glucose, resulting in 7.8% increase in comparison to control. The difference was found to be statistically significant (*p*-value: 0.009). 

However, unlike *A. oryzae* and *A. niger*, the ellagic acid was reported to have an antimicrobial effect on other microorganisms, such as *Tricophyton rubrum*, *Candida albicans* [49], and *Staphylococcus aureus* [50]. For filamentous fungi, the inhibitory mechanism was reported as reduced CYP51 (belongs to cytochrome P450 enzyme family) activity and ergosterol in fungi membrane. Insufficient ergosterol level in membrane results in losing membrane integrity and breaking cells [49].

The tolerance of *A. oryzae* and *A. niger* to ellagic acid can be related to their resistance to ellagic acid attack on the CYP450 enzyme family. Intracellular activity of Ascomycota fungi, including the CYP450 family of enzymes, is also known for being responsible for bioconversion of organic micropollutants [44]. The tolerance of the fungi to this compound can also be related to their ability to convert ellagitannins into ellagic acid [51,52]. Huang et al. [52] reported that *A. oryzae* provided 17.7% ellagic acid from a medium containing 4 g/L ellagitannins via ellagitannin acyl hydrolase enzyme that the fungi produce. Similarly, Ascacio-Valdés et al. [51] stated that ellagitannin in pomegranate was degraded by *A. niger,* and ellagic acid was produced.

**Figure 3:**
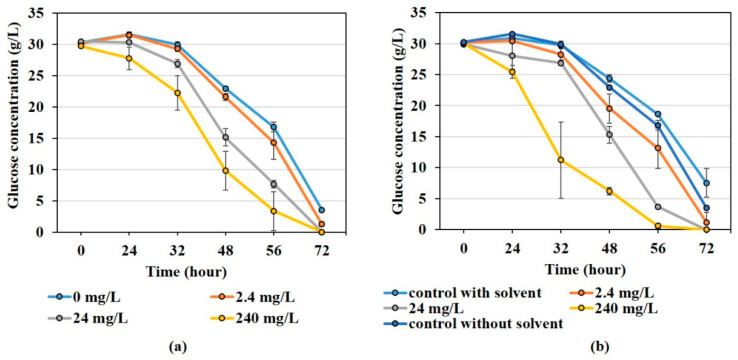
Glucose consumption of *A. niger* in bioactive-compound-added mediums. (**a**) Ellagic acid; (**b**) quercetin.

Additionally, in the control, sugar was not completely consumed by *A. niger*, leaving 3.5 ± 0.5 g/L sugar after 72 h. However, in case of ellagic acid, glucose consumption was faster. In the lowest concentration, 1.3 ± 0.63 g/L glucose was left, while all the glucose was consumed during 72 h at other concentrations. The glucose-consumption trends are shown in Figure 3.

Overall, *A. oryzae* and *A. niger* show great potential for valorization of waste materials, e.g., pomegranate peels, into value-added materials, such as enzymes, organic acids, biomass, etc., without the need of ellagic acid removal, which might add additional cost to the process.

### 3.2. Quercetin

Quercetin, which is extracted from plants and used in food and the pharmaceutical industry [53,54], was tested against *A. oryzae* and *A. niger*. While it did not show any significant effect on *A. oryzae*, the presence of quercetin increased the biomass yield of *A. niger* in the lowest and middle concentrations by 27% and 28%, respectively, in comparison to the control with 0.26 ± 0.04 g biomass/g initial glucose.

This is in opposition to other studies, which reported quercetin as an antimicrobial compound. Hirai et al. [55] reported the inhibition of *Staphylococcus aureus*, a bacteria associated with lipase production [56], in the presence of quercetin at concentration of 50 µM. Rocha et al. [57] mentioned the inhibitory effect that might result in irregularity in nucleic acid synthesis and disorder of mitochondria function.

The resistance of *A. niger* and *A. oryzae* towards quercetin is in accordance with previous studies. These filamentous fungi even have the ability to produce quercetin. *A. niger* has the ability to produce rutinosidase which was extracted and further applied to convert rutin into quercetin by Kapešová et al. [54]. Purewal et al. [58] reported that *A. oryzae* cultivation of pearl millet provided 2.74 mg/g quercetin in the extract obtained from fermented millet, while it was not detected prior to *A. oryzae* cultivation. 

Furthermore, the presence of quercetin increased the glucose-consumption rate of *A. niger*. While there was 7.6 ± 4.6 g/L glucose left in the control after 72 h, in quercetin-containing growth media, all glucose was consumed except the lowest concentration of quercetin, resulting in 1.16 ± 1.65 g/L residual glucose. The higher glucose-consumption rate might be relevant to the activity of glucose transporters or glucose metabolism affected by quercetin. 

*A. oryzae* consumed all glucose within 48 h in all concentrations of quercetin and control; however, a slightly faster consumption trend was observed in the highest quercetin-containing condition. Günata and Vallier [59] stated that beta-glucosidase resistant to glucose inhibition was produced by *A. oryzae* and *A. niger* in the presence of quercetin, with the production ratio of 9% and 3–4%, respectively. Fungal beta-glycosidases are also known to have transglycosylation activity depending on the chemical environment in the medium [60]. It might be interesting to investigate further if quercetin cause such an activity, which is not the focus of this study. 

While bacterial processes are susceptible to quercetin-containing medium, FPW can be valorized via filamentous fungi. Edible biomass production of *A. oryzae* could provide functional food, while industrial applications of *A. niger,* such as enzyme production, could be performed more efficiently. 

### 3.3. Epicatechin 

Epicatechin is one of the polyphenols that has health-improving effect in various aspects, such as coronary and brain health [61] and cancer [62]. In this study, both of the fungi were resistant to the presence of epicatechin. Both *A. oryzae* and *A. niger* were not affected significantly by the presence of epicatechin in the cultivation medium. Supporting this result, in another study, no negative effect of epicatechin–*A. oryzae* interaction on the fungus was reported [63]. Kim et al. [63] reported that during *A. oryzae* fermentation of green tea, some of the phenolic compounds existing in green tea, including epicatechin derivatives, such as epigallocatechin gallate and epicatechin gallate, were converted. Besides, the content of other relevant compounds, such as epicatechin, gallotechin, and epigallocatechin, were enhanced.

On the other hand, unlike these results, epicatechin was reported to have an inhibitory effect on some microorganisms. Some *E. coli* strains were also inhibited by epicatechin when the MIC was less than 20 ppm [64]. The inhibition occurs by breaking cell membranes and preventing the transfer of substances through the membrane [25]. However, in the case of *A. oryzae* and *A. niger,* the fungi seem to be resistant to any negative effect on the cell membrane caused by epicatechin.

On the other hand, in another study conducted by Kanwal et al. [65], derivatives of epicatechin and quercetin showed inhibitory effect against *A. niger*. The growth of fungus was inhibited by (−)-epicatechin-3-*O*-β-glucopyranoside, (−)-epicatechin(2-(3,4-dihydroxyphenyl)-3,4-dihydro-2H-chromene-3,5,7-triol. and quercetin-3-*O*-α-glucopyranosyl-(1→2)-β-d-glucopyranoside. Overall, the fungi were resistant to medium containing epicatechin, which allows them to be used in fruit-waste valorization without any additional steps to remove epicatechin.

### 3.4. Betanin 

Betalains are natural pigments existing in plants. There are two kind of betalains: betaxanthins giving yellow color and betacyanins giving red-violet color. The different kinds of betacyanins are described in [66,67]. In this study, betanin existing in pitahaya, redbeet, tubers, etc., was used. Betanin is widely used as a food additive and colorant and possesses antioxidant properties [68]. In this study, betanin did not cause any inhibition on *A. niger*, while only the middle concentration slightly inhibited *A. oryzae*. The decrease in biomass yield (13%) from 0.21 g biomass/g initial glucose was found to be statistically significant (*p*-value = 0.04). It is interesting that the fungi are resistant to this compound since it was reported to carry some antimicrobial properties, such as inhibiting growth of *Staphylococcus aureus* and *E. coli* in previous studies [69]. The knowledge about the inhibitory mechanism of betanin is scarce in literature. It was reported that betalains bonding with Fe^+2^, Ca^+2^, and Mg^+2^ ions results in preventing bacteria to access them and leading to death due to the lack of necessary substances [70]. Since these fungi can tolerate betanin in the cultivation medium, they are potential candidates for waste valorization of fruit and vegetable residues, such as beetroot, pitahaya, tubers, etc.

### 3.5. Ascorbic Acid

Ascorbic acid, known as vitamin C, exists in wide variety of fruits. In this study, ascorbic acid with maximum 240 mg/L concentration did not show any inhibitory effect on *A. oryzae* and *A. niger*. Furthermore, ascorbic acid at its maximum concentration increased the biomass yield of *A. niger* by 16% in comparison to control with 0.28 ± 0.01 g biomass/g initial glucose. Slightly faster glucose-consumption rate was observed in ascorbic-acid-containing medium compared to control. However, unlike *A. oryzae* and *A. niger*, various industrially important microorganisms are sensitive to ascorbic acid [71]. *Escherichia coli,* a microorganism that is used in the industry due to its rapid growth, can produce various biochemicals thanks to widespread genetic engineering applications [72]. Hence, it is a well-studied microorganism, including waste valorization, such as valorization of lignin [73] and food-waste volatile fatty acids (VFA) [74]. Tajkarimi and Ibrahim [71] stated that combination of ascorbic acid and lactic acid (0.2% and 0.2%, respectively) showed strong inhibition of *E. coli* growth. Similarly, *Pseudomonas* bacteria is also not suitable due to its sensitivity towards coexistence of ascorbic acid (1.71%)/sodium ascorbate (1.98%) pH 4.70 and ellagic acid (0.03%) [75]. The inhibitory effect of ascorbic acid can be because of its oxygen binding property, which prevents bacteria from accessing oxygen [71]. On the other hand, why aerobic *A. oryzae* and *A. niger* were not negatively affected is unknown. The resistant nature of *A. oryzae* is in accordance with Purewal et al. [58] since it was reported that *A. oryzae* fermentation increased the ascorbic acid content of pearl millet from 1.55 mg/g to 10.23 mg/g. 

Ascorbic acid, vitamin C, commonly exists in various fruit and vegetables and their industrial residuals. Producing value-added materials from these wastes can be challenging in the case of using microorganisms susceptible to them. However, *A. oryzae* and *A. niger* would be a good fit since they can grow on materials with a natural concentration of ascorbic acid. 

### 3.6. Limonene

D-limonene is one of the main components in citrus-peel essential oil [76]. It was found to possess antibacterial properties [25,77]. In general, fungi are stated to be more tolerant than bacteria against citrus essential oil [78]. In this study, *A. oryzae* and *A. niger* were found to be resistant up to 240 mg/L limonene. In contrast to this result, in another study, it was reported to be inhibitory for *A. niger* in the agar diffusion method [77]. However, the concentration and growth condition might be the reason for the different result. Marei et al. [79] reported that *A. niger* growth was inhibited by (S)-limonene when EC_50_ was 38.04 mg/L. The difference in the results is possibly because of the different effects of the two isomers on *A. niger*. Nevertheless, D-limonene is the limonene found in citrus essential oil, while S(−)-limonene (L-limonene) is found in trees and herbs [80]. The knowledge about the inhibitory effect of monoterpenes is scarce. A few studies mentioned their effect on enzymes in cell membrane and in altering the fatty acid content of membrane. They may also negatively affect the respiration and membrane permeability [79]. Similarly, Wilkins et al. [81] suggested D-limonene disturbs the cell membrane, which causes the cell content to release and the disorder of H+ and K+ transport system, which is energized by glycolysis.

While some microorganisms, such as some Pseudomonas and *E. coli* strains and some *Aspergillus* strains, are capable of degrading limonene, growth of some microorganisms are inhibited [80]. Ruiz and Flotats [78] mentioned that in anaerobic digestion with organic loading rate higher than 192 mg citrus essential oil/L digester/day, inhibition occurs. Citrus essential oil, which contains up to 90% D-limonene, should be removed from the feedstock prior to the bioprocessing [78]. On the other hand, limonene-resistant microorganisms can provide a less costly alternative since a pretreatment process is not needed. *A. oryzae* and *A. niger* have potential for further utilization of D-limonene containing fruit waste for production of biomass, enzymes, organic acids, etc. 

### 3.7. Car-3-ene

*A. niger* was found to be resistant to this compound in all concentrations tested. On the other hand, the middle concentration of car-3-ene caused 8.8% decrease in *A. oryzae* biomass yield, while the highest concentration increased the biomass yield by 8.1% comparing to 0.21 ± 0.012 g biomass/g initial glucose in control medium. Similarly, in another study, car-3-ene at 1000-ppm concentration caused little increment of biomass growth of *Aspergillus flavus* [82,83], while according to Cosentino et al. [82], car-3-ene (delta-3-carene) inhibited growth of *Aspergillus flavus*.

Both of the fungi were resistant to this compound although it was reported to have antimicrobial effect in literature, e.g., on *Saccharomyces cerevisiae* and *Candida utilis* [84]. An et al. [85] reported little mycelial growth inhibition of *A. niger* due to 1.6 µL/mL car-3-ene using agar diffusion method. Although the concentration is less than our study, the difference might be because of the cultivation method. Although the inhibitory mechanism of car-3-ene is unclear, the effect of terpenoids were interpreted to be damaging the cell membrane by lipophilic compounds [86]. Since the car-3-ene concentration existing in fruit/fruit-processing waste was not found to be inhibitory on *A. niger* and *A. oryzae,* the waste/residual materials can be utilized for production of enzymes that can be used in various industry and organic acids. Edible fungal biomass produced by *A. oryzae* has potential to be used as human food and animal feed.

### 3.8. Myrcene

Myrcene did not cause any significant change of *A. niger* growth, while the highest and middle concentrations caused a slight decrease in biomass yield of *A. oryzae* by 16% and 17%, respectively. Unlike *A. niger* and similar to the slight effect on *A. oryzae,* myrcene was reported to have some inhibitory effect in some previous studies. Less than 0.005% myrcene was stated to be MIC_50_ in anaerobic digestion [27]. Although the information about the inhibitory mechanism of myrcene is scarce, it can be related to the action of monoterpenes with lipophilic content interpreted to be on cell membrane [86]. The resistance of fungi can be due to the difference of cell membrane structure. Besides, the tolerance of *A. niger* can be related to its capability to convert myrcene into 2-methyl-6-methylen-7-octene-2,3-diol, 6-methyl-2-ethenyl-5-heptene-l,2-diol, and 7-methyl-3-methylen-6-octene-l,2-diol [87]. Similarly, another filamentous fungi, *Pleurotus ostreatus,* is reported to convert myrcene into perillene [88]. 

Two common fruit involved in producing processed industrial waste, orange and mango, contain myrcene. The utilization of the residuals in bacterial processes is problematic due to the strong inhibitory effect on myrcene. The removal of the compound by leaching [32] or co-digestion of material together with another waste stream to reduce the concentration of the inhibitory compounds can be applied. On the other hand, another solution can be valorizing the stream without extracting the substance. Since the concentration of myrcene found in fruit residuals (up to 72 μg/L) is lower than the slightly inhibiting concentrations found in this study, filamentous fungi can be used to produce various industrially valuable metabolites and biomass from fruit-processing waste.

### 3.9. Octanol

Octanol showed the strongest inhibitory effect among the flavor compounds tested. The highest concentration of octanol inhibited the fungal growth of *A. oryzae* and *A. niger.* The former had 77%, and the latter had nearly 100% decrease in biomass yield, respectively. In *A. niger* cultivation medium, only a weak cloud was observed; no biomass could have been detected in the sieve during harvest of biomass. While the other concentrations tested showed no significant difference on fungal growth of *A. niger*, 12% of decrease on biomass yield was observed for *A. oryzae* when the octanol concentration was 24 mg/L. The results are in accordance with previous studies, where octanol was reported to be fungistatic for other fungi. Fujita et al. [89] stated the inhibitory effect of octanol on *S. cerevisiae*. The inhibition occurs due to the escalated plasma membrane fluidity of spheroplast form. 

The compound can be used as natural inhibitor for processes in which fungal activity needed to be inhibited, in particular *A. oryzae* and *A. niger*. If the FPW or another substrate containing octanol is the raw material of a process, octanol concentration should be below 240 mg/L. In that case, a pretreatment strategy should be employed, such as leaching, membrane bioreactors, etc.; or mixed substrate can be used in order to reduce the concentration of inhibitory compound.

### 3.10. Hexanal

Existence of hexanal at different concentrations resulted in different effects on both fungi tested. While *A. oryzae* was not affected significantly, the highest concentration of hexanal in *A. niger* cultivation enhanced the biomass yield by 6% in comparison to the lowest concentration of hexanal. The small change was statistically considerable (*p*-value = 0.034). Unlike *A. oryzae* and *A. niger*, hexanal was reported to have antimicrobial effect in some studies. Setzer et al. [90] stated that the hexanal showed inhibitory effect on various microorganism, including *A. niger*. However, the concentrations that were tested were higher than the concentration in this study, which is according to the concentration in fruits, as they exist in trace amounts. Li et al. [91] stated the inhibitory effect of hexanal, as it affects the cell membrane formation and mitochondrial function negatively in *A. flavus*. Hexanal exists in various fruits, such as mango and strawberry. Therefore, it is suggested to valorize the aforementioned fruit residues using *A. niger* and *A. oryzae,* which are resistance to hexanal.

## 4. Conclusions

Octanol, found to be a strong inhibitor for both *A. oryzae* and *A. niger*, has potential to be used as a natural inhibitor. Quercetin and ellagic acid have potential to be used as growth inducers in *A. niger* research and industry. Besides, *A. oryzae* has shown to be appropriate for polyphenol-, ascorbic-acid-, and hexanal-containing waste valorization. Both fungi have potential to be used for the material containing the terpenoids tested in the 0–240 mg/L concentration range; only *A. oryzae* is more efficient below 24 mg/L myrcene, closer to its natural concentration in fruit.

## Figures and Tables

**Table 1:** Solvent concentrations in 0.5% (*w*/*v*) Flavor compound solutions.

Bioactive Compound	Solvent
Octanol	MiliQ water containing 0.5% (*v*/*v*) Tween 80
Ellagic acid	0.5 M NaOH in MiliQ water
(−)-Epicatechin	10% (*v*/*v*) ethanol in MiliQ water
Quercetin	4.7% (*v*/*v*) Ethanol in MiliQ water
Betanin	MiliQ water
Ascorbic Acid	MiliQ water
Limonene	4.4% (*v*/*v*) ethanol in MiliQ water
Hexanal	MiliQ water containing 0.5% (*v*/*v*) Tween 80
Car-3-ene	MiliQ water containing 0.5% (*v*/*v*) Tween 80
Myrcene	MiliQ water containing 0.5% (*v*/*v*) Tween 80

**Table 2:** Statistical analysis results showing significant difference between the effect of different concentrations of each bioactive compound on *A. oryzae* biomass yield.

Bioactive Compound	Concentration (mg/L)	*p*-Value	g Biomass/g Initial Glucose
Betanin	0	0.04	0.21 ± 0.01 ^a^
	2.4		0.20 ± 0.00 ^ab^
	24		0.19 ± 0.02 ^b^
	240		0.20 ± 0.01 ^ab^
Car-3-ene	0	0.003	0.21 ± 0.01 ^b^
	2.4		0.20 ± 0.00 ^bc^
	24		0.19 ± 0.01 ^c^
	240		0.23 ± 0.00 ^a^
Myrcene	0	0.005	0.21 ± 0.01 ^a^
	2.4		0.18 ± 0.01 ^b^
	24		0.18 ± 0.01 ^b^
	240		0.17 ± 0.01 ^b^
Octanol	0	0.000	0.21 ± 0.01 ^a^
	2.4		0.20 ± 0.00 ^ab^
	24		0.19 ± 0.00 ^b^
	240		0.05 ± 0.02 ^c^

Different letters show the significance of difference within each bioactive compound category.

**Table 3:** Statistical analysis results showing significant difference between the effect of different concentrations of each bioactive compound on *A. niger* biomass yield.

Bioactive Compound	Concentration (mg/L)	*p*-Value	g Biomass/g Initial Glucose
Ascorbic Acid	0	0.005	0.28 ± 0.01 ^b^
	2.4		0.29 ± 0.00 ^b^
	24		0.30 ± 0.01 ^b^
	240		0.33 ± 0.01 ^a^
Ellagic Acid	0	0.009	0.28 ± 0.00 ^bc^
	2.4		0.28 ± 0.01 ^c^
	24		0.30 ± 0.01 ^ab^
	240		0.30 ± 0.01 ^a^
Quercetin	0	0.015	0.26 ± 0.04 ^b^
	2.4		0.33 ± 0.03 ^a^
	24		0.33 ± 0.01 ^a^
	240		0.30 ± 0.00 ^ab^
Hexanal	0	0.034	0.30 ± 0.00 ^ab^
	2.4		0.29 ± 0.00 ^b^
	24		0.29 ± 0.01 ^ab^
	240		0.30 ± 0.01 ^a^
Octanol	0	0.000	0.30 ± 0.03 ^a^
	2.4		0.31 ± 0.00 ^a^
	24		0.32 ± 0.02 ^a^
	240		0.00 ± 0.00 ^b^

Different letters show the significance of difference within each bioactive compound category.

## Data Availability

Not applicable.
